# Clinical Characteristics of Fuchs’ Uveitis Syndrome

**DOI:** 10.4274/tjo.99897

**Published:** 2016-04-05

**Authors:** Pınar Nalçacıoğlu, Pınar Çakar Özdal, Mert Şimşek

**Affiliations:** 1 Yıldırım Beyazıt University Faculty of Medicine, Department of Ophthalmology, Ankara, Turkey; 2 Ulucanlar Eye Training and Research Hospital, Ankara, Turkey

**Keywords:** Fuchs’ uveitis syndrome, intraocular pressure, heterochromia, Cataract, complications

## Abstract

**Objectives:**

To evaluate the clinical and demographic properties of Fuchs’ uveitis syndrome (FUS) in Turkish patients.

**Materials and Methods:**

The medical records of 161 patients with FUS followed in the Uveitis Division of Ulucanlar Eye Hospital between 1996 and 2014 were respectively reviewed. The mean age at diagnosis, sex, the number of affected eyes, follow-up period, clinical findings at presentation, complications during the follow-up period, medical and surgical treatments, and best corrected visual acuity at the initial and final visits were recorded.

**Results:**

The study included 171 eyes of 161 patients diagnosed with FUS. Of the patients, 94 (58.4%) were female and 67 (41.6%) were male. The mean age at presentation was 35.2±11.0 (11-65) years. The mean follow-up period was 23.5±32.8 (2-216) months. Ten (6.2%) patients had bilateral involvement. The most common symptoms at presentation were decreased visual acuity or blurred vision in 63 (39.1%) and floaters in 19 (11.8%) patients. Clinical findings at presentation included diffuse small, round, white keratic precipitates in 128 (74.8%) eyes, anterior chamber reaction in 82 (47.9%), vitreous cells in 122 (71.3%), heterochromia in 47 (27.4%) and iris nodules in 32 (18.7%) eyes. During the follow-up period, elevated intraocular pressure occured in 31 (18.1%) eyes and the most common complication was cataract development (89 eyes, 52.0%).

**Conclusion:**

Heterochromia was observed in 27.4% of patients in our study. However, the diffuse small, round keratic precipitates, low-grade anterior chamber reaction and varying degrees of vitreous reaction are more common clinical characteristics that are helpful in making the diagnosis.

## INTRODUCTION

Fuchs’ uveitis syndrome (FUS) accounts for 1-6% of all uveitis cases.^1,2^ This syndrome is diagnosed based on clinical findings, without any laboratory testing. The clinical features of FUS have been described extensively in many studies.^3,4,5^ However, there are data in the literature indicating that the clinical findings of FUS vary between different populations.^3,6,7,8^ Despite the clinical signs being well known, the incorrect and/or delayed diagnosis of FUS is still a frequent occurrence.

The aim of this study was to evaluate the findings at time of presentation, the clinical and demographic characteristics, medical and surgical approaches used and complications during follow-up in Turkish patients diagnosed with FUS presenting to a reference hospital.

## MATERIALS AND METHODS

Of the 1,084 patients who presented to the Uvea unit of the Ulucanlar Eye Hospital, the medical records of the 161 patients (14.8%) diagnosed with FUS were analyzed retrospectively. FUS diagnosis was based on clinical findings as previously described in the literature.^5,9,10,11^ Accordingly, cases exhibiting typically unilateral, chronic, low-grade anterior chamber reaction with varying degrees of vitreous opacity, widespread small- or medium-sized keratic precipitates (KP) in the corneal epithelium, diffuse iris atrophy and/or heterochromia but without acute exacerbations, posterior synechiae or cystoid macular edema were clinically diagnosed with FUS. All patients’ diagnosis and follow-up visits were conducted in the uvea unit by the same physician (P.Ç.Ö.).

A detailed history was obtained from each patient followed by a thorough ophthalmologic examination. Each follow-up visit included best corrected visual acuity (BCVA) assessment, slit-lamp examination of the anterior segment in both eyes, intraocular pressure (IOP) measurement by Goldmann applanation tonometry, and dilated fundus examination using a 90 diopter (D) lens. Patients with IOP ≥21 mmHg underwent angle assessment using gonioscopy. Glaucoma was defined as IOP ≥21 mmHg with optic disc cupping and/or glaucomatous visual field loss, or as the presence of glaucomatous visual field loss despite IOP <21 mmHg.

In order to aid differential diagnosis, erythrocyte sedimentation rate, whole blood count, tuberculin skin test, chest radiograph, angiotensin converting enzyme test, syphilis serology, and cranial magnetic resonance imaging (MRI) were performed as necessary. Fundus fluorescein angiography (FFA) was done in cases with retinal vasculitis findings. Visual field evaluation and ultrasonography were also conducted in selected patients when necessary.

Patients who had sight-limiting KP and cells and were scheduled for surgery were treated with topical corticosteroid for one week prior to the procedure. Patients with severe vitreous haze that significantly limited their vision were treated with posterior sub-Tenon’s triamcinolone injection prior to planning the surgical approach. 

Analysis included patients’ age at diagnosis, gender, clinical findings at disease onset, follow-up duration, systemic diseases, BCVA at initial and final visits, complications, and medical and surgical treatments.

Data were analyzed with Statistical Package for the Social Sciences version 22.0 (SPSS Inc., Chicago, IL, USA). Mean values and percentages were obtained for analysis.

## RESULTS

The present study included 171 eyes of 161 patients diagnosed with FUS. Ninety-four (58.4%) of the patients were female, 67 (41.6%) were male. Mean age at diagnosis was 35.2±11.0 years (range, 11-65 years). Five patients (3.1%) were under the age of 16. Mean follow-up time was 23.5±32.8 months (range, 2-216 months). Four (2.4%) of the patients had rheumatoid arthritis, 1 (0.6%) had type 1 diabetes mellitus, 1 (0.6%) had epilepsy, and 1 (0.6%) had thyroid disease. The right eye was involved in 84 patients (52.1%) and the left eye was involved in 67 patients (41.6%), while 10 patients (6.2%) had bilateral involvement.

Blurred vision or decreased visual acuity was the most common complaint at presentation (63 patients, 39.1%). Sixty-eight patients (42.2%) had no symptoms, and the condition was noticed incidentally during routine examinations in the outpatient clinic. Symptoms at presentation are summarized in Table 1.

BCVA at the initial visit was ≥0.6 in 98 eyes (57.3%), between 0.2 and 0.5 in 38 eyes (22.2%), and ≤0.1 in 35 eyes (20.4%). At the final visit, BCVA distribution was ≥0.6 in 137 eyes (80.1%), between 0.2 and 0.5 in 15 (8.7%), and ≤0.1 in 19 (11.1%). Of the patients with a final BCVA ≤0.1, 1 eye (0.6%) was aphakic, while glaucomatous optic atrophy was observed in 4 eyes (2.4%), cataract in 8 (4.9%), cataract plus vitreous condensation in 4 (2.4%), and vitreous condensation alone in 2 eyes (1.2%).

**Figure 1:**
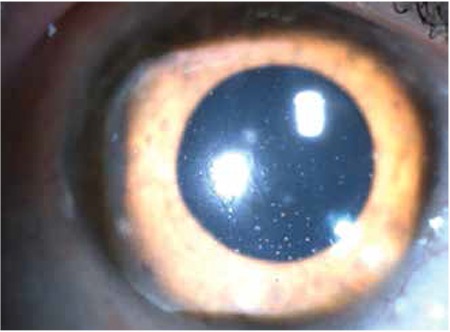
Diffuse, medium-sized, white, round keratic precipitates in a case of Fuchs’ uveitis syndrome

**Figure 2:**
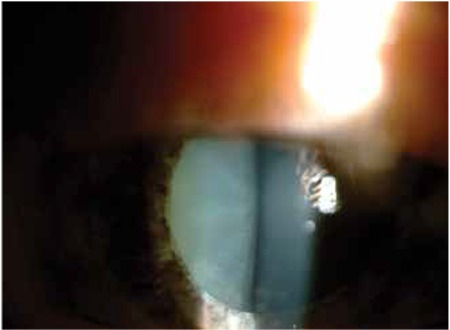
Iris atrophy is more pronounced in the pupillary margin

**Figure 3:**
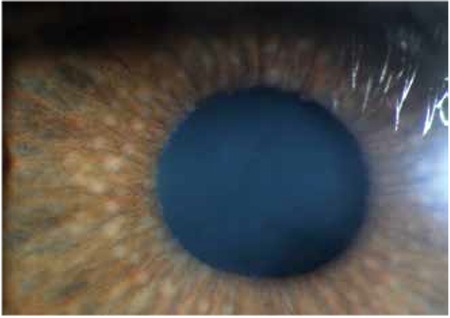
Koeppe nodules at the pupillary border and Busacca nodules in the iris stroma in a Fuchs’ uveitis syndrome patient

KP was observed in 168 eyes (98.2%) at initial presentation, while 3 eyes (1.8%) did not exhibit KP. During follow-up, KP occasionally disappeared and reappeared or fluctuated in severity. In the majority of cases (143 eyes, 85.1%) KP were small to medium-sized, round, thin, white precipitates diffusely scattered over the entire posterior corneal surface (Figure 1). At initial visit the anterior chamber reaction was usually mild to moderate (reaction ≤ [1+] in 67 eyes [39.2%]). Although the severity of vitreous cells and opacity could not be evaluated in some of the involved eyes due to cataract, inflammatory cell reaction between (1+) and (3+) in the vitreous was observed in 120 eyes (70.2%). Forty-seven eyes (27.4%) exhibited heterochromia with varying degrees of iris depigmentation. The iris was atrophic at the pupillary margin in 80 eyes (46.7%), while flattening of iris crypts was observed in 41 eyes (23.9%) (Figure 2). Small multi-focal Koeppe nodules localized to the pupillary margin were present in 32 eyes (18.7%); both Koeppe and Busacca nodules were present in 4 eyes (2.4%) (Figure 3). Posterior synechia was observed in 1 patient (0.7%) who had an IOL implant.

**Figure 4:**
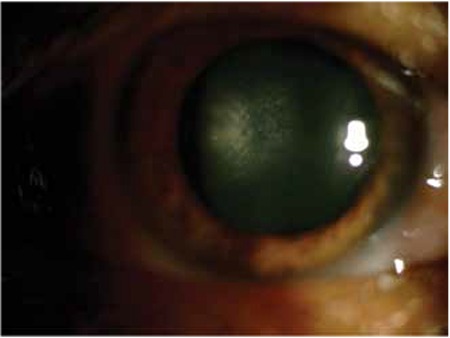
A Fuchs’ uveitis syndrome patient with posterior subcapsular cataract development

At diagnosis, 89 eyes (52%) had cataract. Of these, 2 (2.2%) were nuclear, 5 (5.6%) were mature, and 82 (92.1%) were posterior subcapsular cataract (Figure 4). Twenty-six eyes (15.2%) were pseudophakic. Findings at presentation are summarized in Table 2.

At final visit, 60 eyes (35.0%) were pseudophakic and 1 (0.6%) was aphakic. IOP was within normal limits in 134 patients (83.2%), whereas medical treatment for glaucoma was administered in 31 eyes (18.1%) of 27 patients (16.8%).

The most common complication during follow-up was cataract (89 eyes, 52.0%), followed by glaucoma (31 eyes, 18.1%), vitreous condensation (27 eyes, 15.7%) and secondary cataract (24 eyes, 14.0%). Complications observed are presented in Table 3.

Topical steroid therapy was administered in 26 eyes (15.2%) and periocular steroid injection was administered in 6 eyes (3.5%) due to severe inflammation in the vitreous. Thirty-one eyes (18.1%) received topical antiglaucomatous medication.

The visual acuity of 35 eyes (20.4%) worsened during the follow-up period; these eyes were treated with phacoemulsification (phaco) and intraocular lens (IOL) implantation. Trabeculectomy was performed on 8 eyes (4.7%) with uncontrolled IOP despite maximum medical treatment. Posterior capsule opacification developed in 34 eyes (19.8%) and was treated with YAG laser capsulotomy. Pars plana vitrectomy was performed in a total of 3 eyes (1.8%), 2 (1.2%) due to severe vitreous condensation and 1 (0.6%) due to vitreous hemorrhage. All surgical procedures performed are summarized in Table 4.

## DISCUSSION

FUS, which was first described in 1906 by Fuchs,^4^ cannot be diagnosed by any laboratory test; its diagnosis is based solely on clinical findings. Despite these clinical findings being well defined in many studies, an accurate diagnosis is often delayed.^9,12^ Misdiagnosis results in unnecessary tests and ineffective treatment.^5,7,9,10,11,12,13^ The condition is usually unilateral, with only 5-10% of cases showing bilateral involvement.^10,14^ One of the classic findings is KP, which have been described as diffuse, small, nonpigmented stellate precipitates that are usually nongranulomatous and tend not to aggregate. The vast majority of our patients (93.7%) exhibited unilateral involvement with small to medium white KP diffusely scattered over the corneal endothelium as well as mild anterior uveitis. Tugal-Tutkun et al.^10^ described most of the KPs in their study (74.6%) as medium-sized. Descriptions of the clinical features of FUS have focused on findings related to anterior uveitis, while inflammatory findings in the posterior segment were assigned less importance.^7,9,13,15,16^ However, this plays a major role in the misdiagnosis of FUS. Failure to realize that heterochromia, described as a primary clinical sign of FUS, does not occur in all cases or that inflammatory reaction in the vitreous is a sign of FUS has been reported as the main causes of misdiagnosis.^12,17^ Consistent with these reports, in the current study heterochromia was present in 27.4% of cases at presentation, while inflammatory reaction in the vitreous was observed in 71.3% of cases. Bouchenaki and Herbort^17^ reported that among 105 FUS patients, 77.1% with posterior segment manifestation had been referred with incorrect diagnoses (intermediate uveitis, 56.8%; posterior uveitis, 8.1%; panuveitis, 12.2%) and that their diagnosis were delayed by 3 years on average. Various studies have reported this diagnostic delay ranging from 3 to 6.7 years.^9,12^ The clinical and demographic characteristics of studies in the literature are summarized in Table 5.

The most common complaint at presentation among the patients in the current study was decreased visual acuity or blurred vision (39.1%). Similarly, Yang et al.^3^ reported that decline in visual acuity or blurred vision were the most common symptoms (in 82.6%) of the patients in their study. A large proportion of our patients had no additional symptoms (42.2%) and FUS was detected incidentally during routine outpatient follow-up visits. This is attributable to the disease course characterized by chronic, low-grade inflammation.

FUS usually manifests unilaterally, though the reported rate of bilateral involvement varies in the literature (0-21%).^3,6,7,10^ In the current study, both eyes were involved in 6.2% of our cases. Norrsell and Sjödell^12^ found that patients with bilateral involvement had more progressive disease, developed glaucoma more frequently, and required surgical approaches such as pars plana vitrectomy and cataract surgery more often. Of the bilateral FUS cases in our study, 2 developed glaucoma and another 2 formed epiretinal membrane associated with posterior segment involvement.

Iris changes are a typical finding of FUS. Hypochromia in the affected eye resulting from diffuse pigment loss is the key feature of FUS.^4,7,18,19^ Heterochromia, characterized by color differences between the two eyes, is more apparent in light colored eyes than in dark eyes; therefore, the reported frequency of heterochromia varies widely between populations (12.7-82%).^3,5,6,7,10,12,13,18^ In this study, we found heterochromia at a rate of 27.4%. This finding has long been considered a principal sign of FUS and even lead to it being called ‘Fuchs’ heterochromic iridocyclitis’. However, due to its varying rate of presentation it is important to remember, especially during diagnosis, that heterochromia is not observed in all cases.

Other findings of FUS include iris edema, iris nodules, abnormal iris blood vessels, and more rarely peripheral anterior adhesions and filiform hemorrhage of the anterior chamber angle during paracentesis.^20^ Tugal-Tutkun et al.^10^ analyzed a large case series and emphasized that medium-sized round KP and iris nodules were more common findings than heterochromia in the Turkish population. They observed iris nodules in 32% of the cases in their study, compared to 21% in our study population. This low rate may be due to these nodules, which are small and few in number in the majority of cases, not being recorded.

Many studies have emphasized cataract development as the most common complication observed in FUS patients.^3,7,10,16,19 Tugal-Tutkun et al.10^ found a 56% risk of cataract formation in patients not receiving steroid treatment over their 8-year follow-up period. Yang et al.^3^ also emphasized cataract as the most common (70.7%) complication in their study. Similarly, cataract development was the most common complication observed in our study, at 52%. The variation reported in different studies may be related to disease duration and the chronic nature of the disease. Cataract develops due to changes in lens permeability resulting from recurrent uveitis attacks.21 Unnecessary steroid therapy also increases the risk of cataract formation.

Today, successful visual outcomes can be achieved with modern cataract surgical techniques and IOL implantation. The most common surgical approach utilized during follow-up in our study was phaco-IOL implantation (20.4%). Following cataract surgery, 85.2% of the patients had a final BCVA of 0.6 or better.

Glaucoma is another of the main complications seen in FUS. Its reported frequency varies widely in the literature (11-59%).^3,7,9,16,18^ Glaucoma was detected in 18.1% of our cases. IOP could not be controlled with medical treatment in 25.8% of those patients, necessitating trabeculectomy. IOP was controlled postoperatively with or without medication in all patients who underwent surgery.

There are reports in the literature of posterior segment findings in FUS patients such as chorioretinal scars associated with ocular toxoplasmosis infection, epiretinal fibrosis, and peripheral vascular changes.^7,9,18^ Posterior segment findings observed in the current study included chorioretinal scar (2.3%), peripheral vascular sheathing (2.3%) and intravitreal hemorrhage (0.6%).

## CONCLUSION

In this study we investigated clinical findings in FUS patients. Most of our patients exhibited diffuse, small to medium, white, round or large, stellate KP, low-grade anterior chamber reaction, and vitreous cells and/or vitreous opacity and/or vitreous degeneration with no marked involvement of the posterior pole. We found that vitreous involvement and KP pattern were more prominent diagnostic features than heterochromia. The most common complications during follow-up were cataract, posterior capsule opacification after cataract surgery, glaucoma and vitreous condensation. Based on our data, we believe that a diagnosis of FUS should be considered in cases that are generally unilateral with no marked iris depigmentation but with diffuse small white KP and low-grade anterior chamber reaction, where the fundus is visible and there are no other inflammatory findings except vitreous cells, opacity and/or changes in the vitreous collagen fibers.

## Ethics

Ethics Committee Approval: It was taken from Yıldırım Beyazıt University Yenimahalle Research and Training Hospital,Informed Consent: It was taken.

Peer-review: Externally peer-reviewed.

## Figures and Tables

**Table 1:**
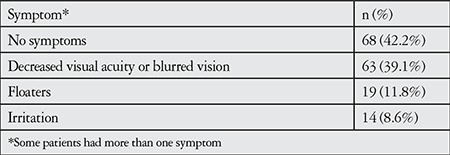
Patients’ symptoms at presentation

**Table 2:**
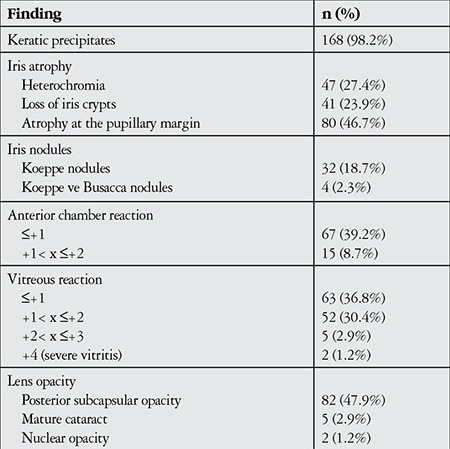
Ocular findings in 171 eyes of 161 patients at time of presentation

**Table 3:**
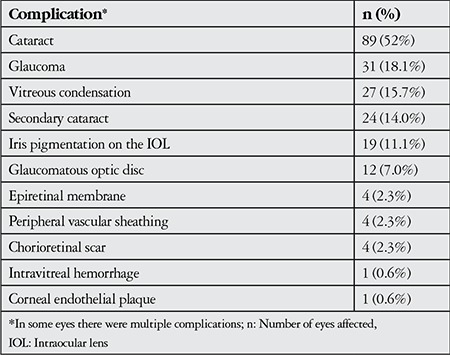
Complications observed in patients with Fuchs’ uveitis syndrome

**Table 4:**
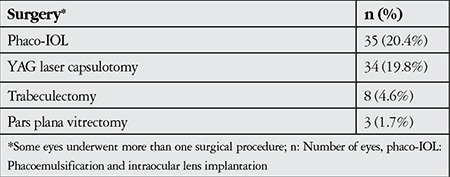
Surgical procedures performed in patients with Fuchs’ uveitis syndrome

**Table 5:**
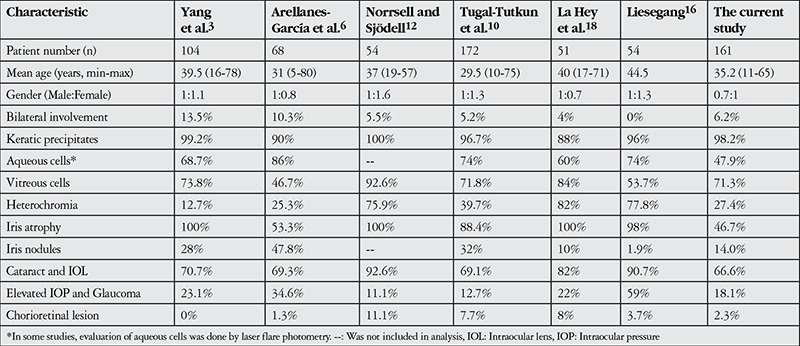
Demographic and clinical characteristics of patients with Fuchs’ uveitis syndrome as reported in the literature
